# Clinical Characteristics and Patterns of Immune Responses in COVID-19 Patients From a Rural Community Hospital

**DOI:** 10.7759/cureus.61600

**Published:** 2024-06-03

**Authors:** Ninh M La-Beck, Young R Lee, Jalpa Patel, Hui Yang, Michal Stout, Alanna Kologey, Amanda Ruesewald, Carlos A Alvarez

**Affiliations:** 1 Department of Immunotherapeutics and Biotechnology, Jerry H. Hodge School of Pharmacy, Texas Tech University Health Sciences Center, Abilene, USA; 2 Center of Excellence in Real-World Evidence, Jerry H. Hodge School of Pharmacy, Texas Tech University Health Sciences Center, Dallas, USA; 3 Ben and Maytee Fisch College of Pharmacy, University of Texas at Tyler, Tyler, USA; 4 Department of Pharmacy Practice, Jerry H. Hodge School of Pharmacy, Texas Tech University Health Sciences Center, Dallas, USA

**Keywords:** elderly, sex differences, immune response, rural community, clinical course, demographics, immunological biomarkers, case series, sars-cov-2, covid-19

## Abstract

Background

Although demographic and clinical factors such as age, certain comorbidities, and sex have been associated with COVID-19 outcomes, these studies were largely conducted in urban populations affiliated with large academic medical centers. There have been very few studies focusing on rural populations that also characterize broader changes in inflammatory cytokines and chemokines.

Methodology

A single-center study was conducted between June 2020 and March 2021 in Abilene, Texas, USA. Patients were included if they presented to the hospital for treatment of COVID-19, had extra biological materials from routine care available, and were between the ages of 0 to 110 years. There were no exclusion criteria. Patient characteristics, symptom presentation, and clinical laboratory results were extracted from electronic health records. Blood specimens were analyzed by protein microarray to quantitate 40 immunological biomarkers.

Results

A total of 122 patients were enrolled, of whom 81 (66%) were admitted to the general non-critical inpatient unit, 37 (30%) were admitted to the intensive or critical care units, and four (3.2%) were treated outpatient. Most hospitalized COVID-19 patients in this rural population were elderly, male, obese, and retired individuals. Predominant symptoms for non-critical patients were shortness of breath, fever, and fatigue. Ferritin levels for outpatient patients were lower on average than those in an inpatient setting and lactate dehydrogenase (LDH) levels were noted to be lower in non-critical and outpatient than those in the intensive care unit setting. Inflammatory biomarkers were positively correlated and consistent with inflammatory cascade. Interleukin (IL)-10 was positively correlated while platelet-derived growth factor was negatively correlated with inflammatory biomarkers. Patients ≥65 years had significantly higher levels of LDH and seven cytokines/chemokines (granulocyte-macrophage colony-stimulating factor (GM-CSF), interleukin IL-1b, IL-6, IL-10, IL-11, macrophage inflammatory protein (MIP)-1d, and IL-8) while levels of five other immune molecules (intercellular adhesion molecule 1 (ICAM-1), monocyte chemoattractant protein 1 (MCP-1), tissue inhibitor of metalloproteinase 2 (TIMP-2), IL-2, and IL-4) were significantly lower compared to those <65 years. Females had significantly higher levels of LDH and 10 cytokines/chemokines (GM-CSF, IL-1b, IL-6, IL-10, IL-11, IL-15, IL-16, MIP-1a, MIP-1d, and IL-8) while levels of TIMP-2 and IL-4 were significantly lower than male patients.

Conclusions

The clinical characteristics of this rural cohort of hospitalized patients differed somewhat from nationally reported data. The contributions of social, environmental, and healthcare access factors should be investigated. We identified age and sex-associated differences in immunological response markers that warrant further investigation to identify the underlying molecular mechanisms and impact on COVID-19 pathogenesis.

## Introduction

In December 2019, health facilities from Wuhan, China reported pneumonia cases of unknown origin, which soon thereafter were classified as the novel coronavirus disease of 2019 (COVID-19) by the Chinese Center for Disease Control and Prevention [1]. Upon isolation, the new strain of coronavirus was named severe acute respiratory syndrome coronavirus (SARS-CoV-2) [2]. Coronaviruses are single-stranded RNA viruses that can infect and cause illness in humans [2,3]. There are four types associated with mild common cold-like respiratory illness, i.e., 229E, OC43, NL63, and HKU1 [4]. Recently, more pathogenic types have been identified, such as SARS-CoV-1, Middle East respiratory syndrome coronavirus (MERS-CoV)-1, and SARS-CoV-2. The distinct characteristic of SARS-CoV-2 is its highly transmittable nature causing global spread, along with its propensity to cause severe disease. After assessing the spread and severity of the virus, the World Health Organization characterized COVID-19 as a pandemic on March 11, 2020 [5]. Years later, the world continues to deal with new strains of SARS-CoV-2 that could cause the next detrimental wave of hospitalizations and deaths. To date, the United States surpasses other countries and territories with an estimated total of 95 million cases and 1 million deaths [6].

The pathogenesis of SARS-CoV-2 derives from an infiltration of the respiratory system via contact droplets from infected individuals [7]. Upon contact, an incubation period of approximately five days occurs, in which SARS-CoV-2 begins to invade the alveolar epithelial cells of the lungs, eventually eliciting an immune response and respiratory symptoms. SARS-CoV-2-positive patients can have an asymptomatic presentation if the immune system can keep the infection at bay. However, classic symptoms of infection include persistent cough, fever, fatigue, and sometimes gastrointestinal effects such as nausea, vomiting, and diarrhea. The severe infections that lead to hospitalizations and intensive care unit (ICU) admissions are usually associated with acute respiratory distress syndrome (ARDS), septic shock, metabolic acidosis, and coagulopathies that can lead to multiple organ damage and death.

Since the start of the pandemic, much effort has gone into identifying biomarkers associated with worse outcomes, such as ARDS and mortality, in COVID-19-infected patients. The identification of these biomarkers helps with risk stratification models for clinical approaches. The most common hematologic biomarkers shown to be significantly associated with disease progression are neutrophilia, lymphopenia, and thrombocytopenia [8-12]. The most common inflammatory markers significantly associated with disease progression are elevated C-reactive protein (CRP), ferritin, cytokines, interferons, and tumor necrosis factor (TNF) [13]. Other biomarkers that are potential predictors of disease severity, related to coagulopathies and end-organ damage, include elevations in D-dimer, lactate dehydrogenase (LDH), liver function enzymes, troponin, serum creatinine, and blood urea nitrogen. Across studies, the most predictive biomarkers that had significant correlations with mortality that should be closely monitored in COVID-19 patients are white blood count (WBC), lymphocyte count, CRP, ferritin, fragment D-dimer (D-dimer), and interleukin (IL)-6 [14].

In addition to biomarkers associated with the disease, there are patient demographic and genetic factors that may predict patient outcomes. It is now widely accepted that comorbidities, such as cardiovascular disease, diabetes, and chronic obstructive pulmonary disease, are associated with worse prognosis in COVID-19 patients [2]. In addition, factors that influence the ability to mount an adequate immune response, such as increased age and an immunocompromised state, also lead to worse outcomes [15,16]. Moreover, throughout the pandemic, differences in virus susceptibility and disease severity have been observed between male and female sexes [2,17]. Females have been reported to produce a more robust immune response to pathogens due to differences in hormones [18]. However, other mechanisms responsible for the sex disparities seen with SARS-CoV-2 infections have been hypothesized [17]. For instance, the pathogenesis of SARS-CoV-2 involves targeting tissues that express angiotensin-converting enzyme 2 (ACE2), which is a protective regulator of the renin-angiotensin-aldosterone system (RAAS). As ACE2 is X-linked and estrogen has been shown to increase its expression, males may be more at risk for losing these protective regulatory effects of RAAS, which contribute to disease severity.

Most efforts in understanding and addressing the implications of the pandemic have focused on highly populated regions and urban communities. However, rural communities have presented unique challenges that make them more vulnerable to poor outcomes [19-21]. Many of the risk factors previously mentioned are amplified in these areas, such as elderly populations and individuals with underlying comorbid conditions [15,16]. Along with populations with increased risk factors, these rural communities face healthcare and economic inequities that further exacerbate the impact of the pandemic [22,23]. Considering the disparities in rural communities, it is important to target COVID-19 investigations specifically in these areas to not only understand the factors that contribute to COVID-19 outcomes but also to use that information to improve clinical approaches and health policies specific to these areas. The objective of this study is to describe the clinical characteristics of COVID-19 patients in a rural West Texas community and explore age and sex-associated differences in immune responses that can be used to optimize clinical care and public health policies in the rural community setting.

## Materials and methods

We identified patients who were COVID-19 positive at a rural community hospital in Abilene, Texas from June 29, 2020, to March 7, 2021. Patients were included if they presented to the hospital for treatment of COVID-19, were screened for possible COVID-19 diagnosis or confirmed diagnosis by city/county health district or healthcare providers, had extra biological materials from routine screening or clinical care available, and were between the ages of 0 to 110 years. There were no exclusion criteria. This study was approved by the Texas Tech University Health Science Center institutional review boards with a waiver of informed consent.

Patients with COVID-19 infection were verified by testing via polymerase chain reaction. Once confirmed, the clinical lab director or associated personnel sent a list of patients with their clinical blood samples to be retrieved by Texas Tech research personnel, and each subject was assigned a unique study ID code. Once coded, the samples were immediately processed into cellular and plasma components and then stored for analysis of biomarkers. The antibody array for secreted inflammatory cytokines and chemokines was performed with a 40-plex Quantibody Human Inflammation Array 3 kit (QAH-INF-3, RayBioTech) with standard curves for the following molecules: B-lymphocyte chemoattractant (BLC), eotaxin, eotaxin-2, granulocyte colony-stimulating factor (G-CSF), granulocyte-macrophage colony-stimulating factor (GM-CSF), macrophage colony stimulating factor (M-CSF), small inducible cytokine A1 (I-309), intercellular adhesion molecule 1 (ICAM)-1, interferon gamma (IFN-g), IL-1a, IL-1b, IL-2, IL-4, IL-5, IL-6, IL-6 soluble receptor (IL-6sR), IL-7, IL-8, IL-10, IL-11, IL-12 subunit p40 (IL-12p40), IL-12 subunit p70 (IL-12p70), IL-13, IL-15, IL-16, IL-17, macrophage chemoattractant protein 1 (MCP-1), monokine induced by interferon gamma (MIG), macrophage inflammatory protein (MIP)-1a, MIP-1b, MIP-1d, platelet-derived growth factor (PDGF), regulated upon activation, normal T-cell expressed and secreted protein (RANTES), tissue inhibitor of metalloproteinase (TIMP)-1, TIMP-2, TNF-α, TNF-β, TNF receptor 1 (TNF-R1), and TNF receptor 2 (TNF-R2). Clinical data including demographic characteristics, symptom presentation, comorbidities, and clinical laboratory findings were collected from the electronic medical record.

Descriptive statistics were used to summarize patient demographics such as age, sex, race, height, weight, body mass index (BMI), occupation, residence type, and symptoms associated with COVID-19 cases. Statistical analyses were performed using analysis of variance and chi-square tests to compare continuous data and categorical data, respectively. Unadjusted p-values were reported, and a p-value less than 0.05 was considered statistically significant except for the protein microarray data where Bonferroni correction for multiple comparisons was applied and a p-value less than 0.00102 was considered statistically significant. Covariance between age, BMI, monocyte count, and various immunological biomarkers was assessed using the Pearson correlation coefficient. Statistical software used were SAS 9.4 (SAS Institute, Cary, NC, USA) and GraphPad Prism version 9.0 or higher.

## Results

Clinical characteristics

Data and samples were collected from 122 patients. Patient demographics are presented in Table 1. Age, weight, and BMI by sex and race/ethnicity are shown in Table 2. The patients who presented to the hospital ultimately received care in three different settings, distributed as follows: non-critical inpatients, 81 (66%), ICU, 37 (30%), and self-care, four (3.2%). The age (mean ± standard deviation) of non-critical inpatients, ICU patients, and self-care patients were 65.4 ± 16.5, 64.2 ± 15.9, and 57.3 ± 15.0 years, respectively. All patient care settings had a higher prevalence of White patients followed by Hispanic patients who had a BMI greater than 30 kg/m^2^. In addition, 49 (60.5%) non-critical inpatients, 20 (54.1%) ICU patients, and two (50%) self-care patients were male. For occupation status, retired individuals comprised the largest category for patients admitted for treatment, with 35 (43.2%) non-critical inpatients, 16 (43.2%) ICU patients, and one (25%) self-care patient who had retired. Those in the general workforce, such as farmers, construction workers, and other occupations, comprised 22 (27.2%) non-critical inpatients and seven (18.9%) ICU-admitted patients. State school inmates represented six (7.4%) non-critical inpatients and one (25%) self-care patient.

**Table 1:** Patient demographics. Mean with standard deviation shown for continuous variables. ICU: intensive care unit; BMI: body mass index; N/A: not available

	Non-critical inpatient (N = 81)	ICU (N = 37)	Self-care (N = 4)
Age (years)	65.4 ± 16.5	64.2 ± 15.9	57.3 ± 15.0
Sex, N (%)			
Male	49 (60.5)	20 (54.1)	2 (50.0)
Female	32 (39.5)	17 (46.0)	2 (50.0)
Race, N (%)			
Hispanic	21 (25.9)	6 (16.2)	1 (25.0)
White	53 (65.4)	28 (75.7)	3 (75.0)
Black	6 (7.4)	2 (5.4)	0 (0.0)
Other	1 (1.2)	1 (2.7)	0 (0.0)
Height (cm)	168.3 ± 10.3	169.3 ± 10.0	166.4 ± 14.4
Weight (kg)	91.3 ± 27.9	88.4 ± 25.4	90.3 ± 22.7
BMI (kg/m^2^)	32.3 ± 9.7	30.8 ± 8.6	32.5 ± 7.6
Occupation, N (%)			
Retired	35 (43.2)	16 (43.2)	1 (25.0)
Unemployed	5 (6.2)	4 (10.8)	0 (0.0)
Disabled	6 (7.4)	5 (13.5)	0 (0.0)
State school inmate	7 (8.6)	0 (0.0)	1 (25.0)
Others	22 (27.2)	7 (18.9)	2 (50.0)
N/A	6 (7.4)	5 (3.5)	0 (0.0)
Residence, N (%)			
Apartment	5 (6.2)	2 (5.4)	0 (0.0)
House	62 (76.5)	32 (86.5)	2 (50.0)
Nursing home	6 (7.4)	2 (5.4)	0 (0.0)
Prison	3 (3.7)	0 (0.0)	1 (25.0)
Other	5 (6.2)	1 (2.7)	1 (25.0)

**Table 2:** Baseline characteristics by sex and race/ethnicity. SD: standard deviation; BMI: body mass index

	Sex		Race/Ethnicity			
	Male	Female	Hispanic	White	Black	Other
Age (years)						
N	71	51	28	84	8	2
Mean ± SD	64.5 ± 16.4	65.2 ± 16.0	60.2 ± 15.4	67.0 ± 16.5	58.6 ± 14.1	61.0 ± 4.2
Range	25, 97	21, 92	25, 90	21, 97	36, 59	58, 64
Weight (kg)						
N	71	51	28	84	8	2
Mean ± SD	93.1 ± 27.7	86.6 ± 25.4	85.0 ± 22.6	91.0 ± 27.0	107.6 ± 35.5	71.8 ± 4.5
Range	45.4, 173.0	45.0, 152.5	45.9, 82.2	45.0, 173.0	63.3, 165.6	68.6, 75.0
BMI (kg/m^2^)						
N	71	51	28	84	8	2
Mean ± SD	30.7 ± 8.3	33.4 ± 10.5	31.6 ± 9.1	31.7 ± 9.5	35.1 ± 9.0	26.0 ± 6.0
Range	16.7, 59.7	17.3, 59.5	19.8, 59.5	16.7, 59.7	25.1, 47.6	21.7, 30.2

Patients initially were diagnosed with COVID-19 by different tests that included RNA, antibody, and antigen tests. The RNA test was the most used, with 77 (95.1%), 36 (97.3%), and four (100%) non-critical inpatients, ICU patients, and self-care patients receiving this test, respectively. These sequelae and symptoms are described in Table 3. For non-critical inpatients, 39 (48.2%) recovered without sequelae compared to seven (18.9%) ICU patients. For ICU patients, 16 (43.2%) patients died from COVID-19-related causes compared to five (6.2%) non-critical inpatients, and no deaths were reported in self-care patients. Patients who were hospitalized had shortness of breath as their predominant symptom, with 54 (66.7%) non-critical inpatients and 25 (67.6%) ICU patients, while no self-care patients reported shortness of breath. Other symptoms that were prevalent for non-critical inpatients and ICU patients were fever and fatigue. Although cough was a relatively common symptom of COVID-19, it was absent in the majority of patients across all three patient care settings: 47 (58%) non-critical inpatients, 29 (78.4%) ICU patients, and three (75%) self-care patients did not report a cough.

**Table 3:** Symptoms associated with COVID-19 cases. ICU: intensive care unit

	Non-critical inpatient (N = 81)	ICU (N = 37)	Self-care (N = 4)
Sequelae, N (%)			
Recovered without sequelae	39 (48.2)	7 (18.9)	0 (0.0)
Recovered with sequelae	3 (3.7)	4 (10.8)	1 (25.0)
Death related to COVID-19	5 (6.2)	16 (43.2)	0 (0.0)
Death unrelated to COVID-19	1 (1.2)	3 (8.1)	0 (0.0)
Referred to follow-up as outpatient	30 (37.0)	5 (13.5)	3 (75.0)
Outpatient hospice	3 (3.7)	2 (5.4)	0 (0.0)
Symptoms, N (%)			
Fever	33 (40.7)	13 (35.1)	1 (25.0)
Fatigue	30 (37.0)	9 (24.3)	1 (25.0)
Nausea	17 (21.0)	3 (8.1)	2 (50.0)
Loss of smell	5 (6.2)	1 (2.7)	1 (25.0)
Shortness of breath	54 (66.7)	25 (67.6)	0 (0.0)
Vomiting	9 (11.1)	3 (8.1)	1 (25.0)
Diarrhea	22 (27.2)	4 (10.8)	2 (50.0)
Cough			
No	47 (58.0)	29 (78.4)	3 (75.0)
Productive	20 (24.7)	4 (10.8)	1 (25.0)
Non-productive	14 (17.3)	4 (10.8)	0 (0.0)
Headache	8 (9.9)	2 (5.4)	2 (50.0)
Angina	13 (16.1)	2 (5.4)	0 (0.0)
Anorexia	9 (11.1)	3 (8.1)	1 (25.0)
Altered mental status	6 (7.4)	5 (13.5)	0 (0.0)
Myalgias	15 (18.5)	2 (5.4)	1 (25.0)
Chills	13 (16.1)	3 (8.1)	0 (0.0)
Dizziness	5 (6.2)	2 (5.4)	0 (0.0)
Diaphoresis	4 (4.9)	1 (2.7)	1 (25.0)
Sore throat	2 (2.5)	0 (0.0)	0 (0.0)
Abdominal pain	5 (6.2)	0 (0.0)	0 (0.0)
Other	12 (14.8)	5 (13.5)	0 (0.0)

Clinical and molecular biomarkers

Analysis by severity subgroups based on whether they were in non-critical inpatient, self-care, or ICU settings showed no significant differences in the measurements of any clinical laboratory markers between these groups (Table 4); however, there were a few notable trends. Ferritin levels for self-care patients were lower on average than those in non-critical and ICU settings, and LDH levels were lower in non-critical inpatients and self-care patients than those in the ICU setting.

**Table 4:** Laboratory changes associated with COVID-19 cases. SD: standard deviation; CRP: C-reactive protein; LDH: lactate dehydrogenase; D-dimer: fragment D-dimer; WBC: white blood count

	Severity	N	Mean	SD (±)	P-value
CRP (mg/L)	All	113	11.08	10.37	0.4806
	Non-critical	75	10.29	9.77	
	ICU	35	13.08	11.57	
	Outpatient	3	7.59	10.17	
Ferritin (ng/mL)	All	102	1,413.81	3,268.40	0.9742
	Non-critical	66	1,084.70	2,210.17	
	ICU	33	2,189.67	4,774.16	
	Outpatient	3	119.70	68.07	
LDH (U/L)	All	104	676.56	2,555.51	0.3972
	Non-critical	67	336.72	165.47	
	ICU	34	1,388.18	4,421.75	
	Outpatient	3	201.33	30.89	
D-dimer (ng/mL FEU)	All	92	19.80	114.33	0.6984
	Non-critical	60	16.53	111.04	
	ICU	31	26.75	123.76	
	Outpatient	1	0.34	-	
WBC (×10⁶ cells/mm³)	All	122	10.77	6.69	0.6464
	Non-critical	81	10.12	6.30	
	ICU	37	12.48	7.47	
	Outpatient	4	10.028	5.08	
Platelet (×10³ cells/mL)	All	122	237.52	114.03	0.3982
	Non-critical	81	242.62	115.90	
	ICU	37	231.35	115.54	
	Outpatient	4	191.50	45.00	
Neutrophil (×10³ cells/mL)	All	120	76.67	13.30	0.6349
	Non-critical	79	73.87	13.93	
	ICU	37	82.46	9.84	
	Outpatient	4	78.38	13.58	
Lymphocyte (×10³ cells/mL)	All	120	15.07	12.40	0.5403
	Non-critical	79	17.032	11.72	
	ICU	37	11.01	13.05	
	Outpatient	4	13.93	13.50	
Monocyte (cells/mL)	All	120	6.62	3.57	0.1217
	Non-critical	79	6.87	3.60	
	ICU	37	6.07	3.62	
	Outpatient	4	6.70	2.23	

**Figure 1:**
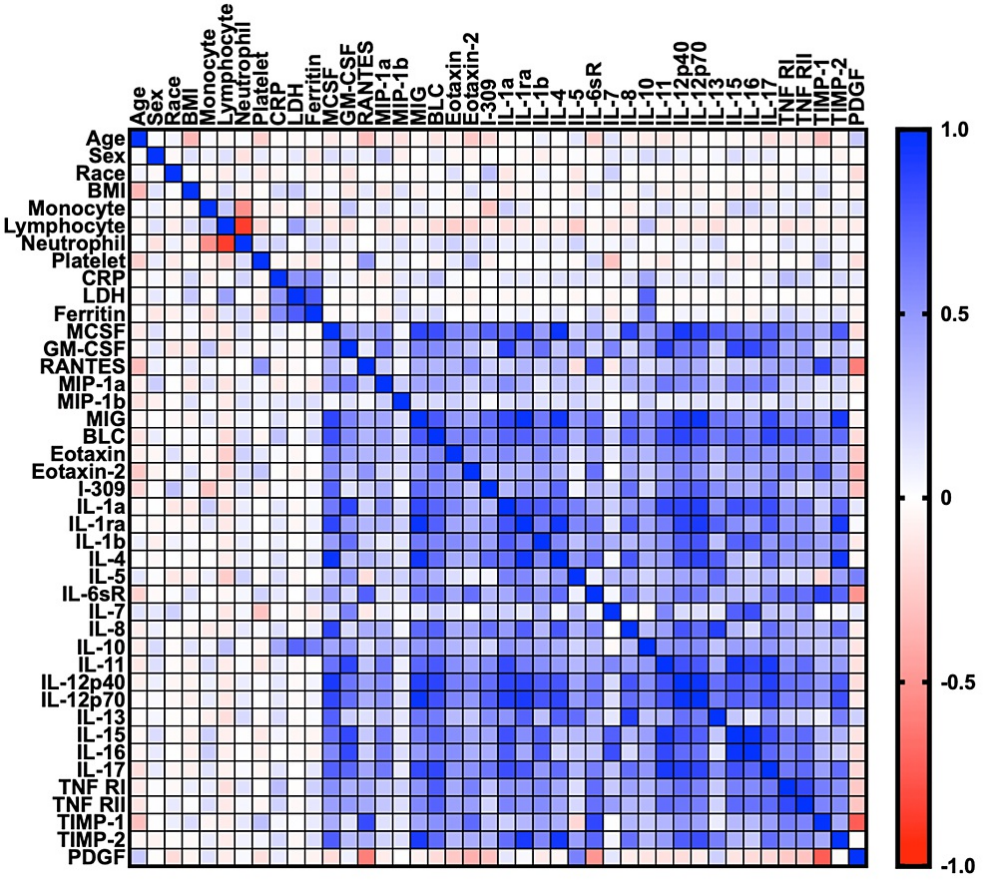
Correlation (Pearson R) between patient factors and immunological biomarkers. BMI: body mass index; CRP: C-reactive protein; LDH: lactate dehydrogenase; D-dimer: fragment D-dimer; BLC: B-lymphocyte chemoattractant; G-CSF: granulocyte colony-stimulating factor; GM-CSF: granulocyte-macrophage colony-stimulating factor; M-CSF: macrophage colony-stimulating factor; I-309: small inducible cytokine A1; ICAM: intercellular adhesion molecule; IFN-g: interferon-gamma; IL: interleukin; IL-6sR: IL-6 soluble receptor; IL-12p40: IL-12 subunit p40; IL-12p70: IL-12 subunit p70; MCP-1: macrophage chemoattractant protein 1; MIG: monokine induced by interferon-gamma; MIP: macrophage inflammatory protein; PDGF: platelet-derived growth factor; RANTES: regulated upon activation normal T-cell expressed and secreted protein; TIMP: tissue inhibitor of metalloproteinase; TNF: tumor necrosis factor; TNF-R1: TNF receptor 1; TNF-R2: TNF receptor 2

Adequate blood specimens for microarray analysis were available for 72 out of the 122 patients. The majority of the immune biomarkers were positively correlated (Figure 1), consistent with an inflammatory cascade response, while PDGF was negatively correlated with the inflammatory markers. IL-10 was positively correlated with the clinical laboratory markers CRP, LDH, and ferritin. Age generally had a negative correlation with blood biomarkers. Analysis by age ≥65 years versus age <65 years showed significant differences between these two groups (Table 5). We found that patients aged ≥65 years had significantly lower levels of LDH and seven cytokines/chemokines (GM-CSF, IL-1b, IL-6, IL-10, IL-11, MIP-1d, and IL-8) while levels of five other immune molecules (ICAM-1, MCP-1, TIMP-2, IL-2, and IL-4) were significantly higher than patients aged <65 years. Analysis by sex showed that females had higher levels of nearly all biomarkers compared to males (Table 6). Female patients had significantly higher levels of LDH and 10 cytokines/chemokines (GM-CSF, IL-1b, IL-6, IL-10, IL-11, IL-15, IL-16, MIP-1a, MIP-1d, and IL-8) while levels of TIMP-2 and IL-4 were significantly lower than male patients.

**Table 5:** Immune biomarkers by age. *: A p-value <0.00102 was considered statistically significant after Bonferroni adjustment for multiple comparisons. SD: standard deviation; CRP: C-reactive protein; LDH: lactate dehydrogenase; D-dimer: fragment D-dimer; WBC: white blood count; BLC: B-lymphocyte chemoattractant; G-CSF: granulocyte colony-stimulating factor; GM-CSF: granulocyte-macrophage colony-stimulating factor; M-CSF: macrophage colony-stimulating factor; I-309: small inducible cytokine A1; ICAM: intercellular adhesion molecule; IFN-g: interferon-gamma; IL: interleukin; IL-6sR: IL-6 soluble receptor; IL-12p40: IL-12 subunit p40; IL-12p70: IL-12 subunit p70; MCP-1: macrophage chemoattractant protein 1; MIG: monokine induced by interferon-gamma; MIP: macrophage inflammatory protein; PDGF: platelet-derived growth factor; RANTES: regulated upon activation normal T-cell expressed and secreted protein; TIMP: tissue inhibitor of metalloproteinase; TNF: tumor necrosis factor; TNF-R1: TNF receptor 1; TNF-R2: TNF receptor 2

Biomarker	Age <65 (mean ± SD) N = 32 (56% male)	Age ≥65 (mean ± SD) N = 40 (55% male)	P-value
Clinical Lab			
LDH (U/L)	997.9 ± 3737	401.1 ± 362.3	<0.0001*
CRP (mg/mL)	11.3 ± 12.1	10.9 ± 8.6	0.012
Monocyte (cells/mL)	6.3 ± 3.0	6.9 ± 4.0	0.0181
D-dimer (ng/mL FEU)	23.2 ± 131.0	16.8 ± 98.7	0.0581
Ferritin (ng/mL)	1,520 ± 3,706	1,323 ± 2,874	0.073
Lymphocyte (×10³ cells/mL)	16.3 ± 13.1	14.0 ± 11.8	0.4074
Neutrophil (×10³ cells/mL)	76.4 ± 12.8	76.9 ± 13.9	0.5278
Platelet (×10³ cells/mL)	249.7 ± 118.4	226.8 ± 109.9	0.5641
WBC (×10⁶ cells/mm³)	10.5 ± 6.7	11.0 ± 6.7	0.9907
Microarray			
GM-CSF (pg/mL)	80.0 ± 282.2	56.8 ± 103.6	<0.0001*
IL-1b (pg/mL)	79.8 ± 394.2	36.0 ± 132.	<0.0001*
IL-6 (pg/mL)	129.6 ± 232.6	66.3 ± 83.9	<0.0001*
IL-10 (pg/mL)	23.6 ± 62.1	9.5 ± 24.9	<0.0001*
IL-11 (pg/mL)	28.8 ± 77.5	11.2 ± 29.1	<0.0001*
MIP-1d (pg/mL)	79,778 ± 433E3	11,990 ± 52,362	<0.0001*
ICAM-1 (pg/mL)	20,036 ± 19,676	32,568 ± 71,416	<0.0001*
MCP-1 (pg/mL)	262.7 ± 292.7	370.9 ± 719.2	<0.0001*
TIMP-2 (pg/mL)	15,709 ± 19,109	16,380 ± 43,086	<0.0001*
IL-2 (pg/mL)	507.6 ± 1,313	1,305 ± 2,641	0.0001*
IL-4 (pg/mL)	45.7 ± 188.0	77.6 ± 373.4	0.0002*
IL-8 (pg/mL)	526.9 ± 1,944	264.5 ± 1,024	0.0002*
TNF-α (pg/mL)	352.1 ± 961.9	723.7 ± 1,722	0.0012
MIP-1b (pg/mL)	55.2 ± 60.1	42.9 ± 35.6	0.0023
IL-15 (pg/mL)	354.9 ± 887.3	232.9 ± 529.0	0.0025
IL-13 (pg/mL)	10.2 ± 33.3	9.8 ± 20.4	0.0043
IL-5 (pg/mL)	79.6 ± 136.1	135.9 ± 224.9	0.0049
M-CSF (pg/mL)	34.1 ± 98.8	42.1 ± 163.2	0.005
IL-16 (pg/mL)	827.5 ± 1,792	565.8 ± 1,130	0.0068
IL-17 (pg/mL)	11.9 ± 22.6	5.5 ± 14.3	0.0078
G-CSF (pg/mL)	208.5 ± 594.9	177.4 ± 933.3	0.0114
IL-1ra (pg/mL)	1,125 ± 2,294	1,280 ± 3,471	0.0195
BLC (pg/mL)	42.4 ± 72.3	28.4 ± 50.1	0.0303
MIG (pg/mL)	450.5 ± 1,437	461.1 ± 2,103	0.0314
MIP-1a (pg/mL)	263.7 ± 457.5	168.4 ± 318.3	0.0327
IFNg (pg/mL)	109.7 ± 219.8	133.5 ± 314.3	0.0427
TNF-β (pg/mL)	3,854 ± 10,466	7,816 ± 14,894	0.0456
I-309 (pg/mL)	139.0 ± 318.9	90.0 ± 232.3	0.0617
IL-1a (pg/mL)	175.1 ± 382.2	164.4 ± 287.2	0.0917
IL-7 (pg/mL)	119.5 ± 306.1	108.7 ± 235.6	0.122
PDGF (pg/mL)	2,378 ± 4,242	4,695 ± 5,537	0.1291
IL-6sR (pg/mL)	3,510 ± 1,277	3,185 ± 1,620	0.174
Eotaxin (pg/mL)	89.3 ± 119.0	65.3 ± 97.6	0.2401
TNF-RI (pg/mL)	7,697 ± 7,427	6,684 ± 6,119	0.2509
IL-12p40 (pg/mL)	261.7 ± 686.2	197.5 ± 568.8	0.2661
TIMP-1 (pg/mL)	85,358 ± 45,863	65,747 ± 54,335	0.3333
TNF-RII (pg/mL)	28,394 ± 23,694	22,395 ± 20,399	0.3734
IL-12p70 (pg/mL)	5.0 ± 16.4	5.6 ± 18.4	0.5207
Eotaxin-2 (pg/mL)	349.7 ± 224.3	260.5 ± 205.4	0.5972
RANTES (pg/mL)	12,162 ± 7,559	9,394 ± 7,539	0.9775

**Table 6:** Immune biomarkers by sex. *: A p-value <0.00102 was considered statistically significant after Bonferroni adjustment for multiple comparisons. SD: standard deviation; CRP: C-reactive protein; LDH: lactate dehydrogenase; D-dimer: fragment D-dimer; WBC: white blood count; BLC: B-lymphocyte chemoattractant; G-CSF: granulocyte colony-stimulating factor; GM-CSF: granulocyte-macrophage colony-stimulating factor; M-CSF: macrophage colony-stimulating factor; I-309: small inducible cytokine A1; ICAM: intercellular adhesion molecule; IFN-g: interferon-gamma; IL: interleukin; IL-6sR: IL-6 soluble receptor; IL-12p40: IL-12 subunit p40; IL-12p70: IL-12 subunit p70; MCP-1: macrophage chemoattractant protein 1; MIG: monokine induced by interferon-gamma; MIP: macrophage inflammatory protein; PDGF: platelet-derived growth factor; RANTES: regulated upon activation normal T-cell expressed and secreted protein; TIMP: tissue inhibitor of metalloproteinase; TNF: tumor necrosis factor; TNF-R1: TNF receptor 1; TNF-R2: TNF receptor 2

	Male (mean ± SD) N = 40 (45% <65 years)	Female (mean ± SD) N = 32 (44% <65 years)	P-value
Clinical lab			
LDH (U/L)	423.1 ± 238.5	1022 ± 3,918	<0.0001*
Platelet (×10³ cells/mL)	227.0 ± 96.8	252.2 ± 134.0	0.0121
Lymphocyte (×10³ cells/mL)	13.5 ± 10.9	17.3 ± 14.1	0.0507
CRP (ng/mL)	10.6 ± 9.3	11.7 ± 11.8	0.08
WBC (×10⁶ cells/mm³)	10.6 ± 6.1	10.9 ± 7.5	0.1064
Neutrophil (×10³ cells/mL)	78.3 ± 12.3	74.4 ± 14.4	0.2229
Monocyte (cells/mL)	6.4 ± 3.3	6.9 ± 3.9	0.23
D-dimer ng/mL FEU)	17.3 ± 111.9	24.3 ± 120.1	0.6283
Ferritin (ng/mL)	1731 ± 3239	941.3 ± 3295	0.8893
Microarray			
GM-CSF (pg/mL)	44.7 ± 91.5	95.1 ± 285.4	<0.0001*
IL-1b (pg/mL)	32.4 ± 128.8	84.3 ± 395.2	<0.0001*
IL-6 (pg/mL)	60.1 ± 77.1	137.2 ± 233.2	<0.0001*
IL-10 (pg/mL)	8.4 ± 25.0	25.1 ± 61.7	<0.0001*
IL-11 (pg/mL)	11.1 ± 26.5	28.9 ± 78.6	<0.0001*
IL-15 (pg/mL)	183.2 ± 399.9	417.0 ± 956.7	<0.0001*
IL-16 (pg/mL)	539.5 ± 920.7	860.3 ± 1,932	<0.0001*
MIP-1a (pg/mL)	128.6 ± 193.1	313.5 ± 524.8	<0.0001*
MIP-1d (pg/mL)	9,338 ± 47,357	83,094 ± 433E3	<0.0001*
TIMP-2 (pg/mL)	17,878 ± 43,485	13,837 ± 17,683	<0.0001*
IL-4 (pg/mL)	73.7 ± 373.8	50.5 ± 187.7	0.0002*
IL-8 (pg/mL)	272.6 ± 1,031	516.8 ± 1,941	0.0002*
MIP-1b (pg/mL)	51.9 ± 57.7	44.0 ± 32.6	0.0015
IL-13 (pg/mL)	8.8 ± 20.1	11.4 ± 33.4	0.0029
ICAM-1 (pg/mL)	26,538 ± 42,287	27,573 ± 68,142	0.0052
M-CSF (pg/mL)	40.0 ± 162.6	36.8 ± 100.1	0.0065
G-CSF (pg/mL)	183.3 ± 932.6	201.0 ± 596.5	0.012
IL-17 (pg/mL)	6.5 ± 14.9	10.7 ± 22.4	0.0178
IL-1ra (pg/mL)	1,326 ± 3,468	1,067 ± 2,296	0.02
MCP-1 (pg/mL)	333.2 ± 659.3	309.9 ± 442.6	0.0244
IL-7 (pg/mL)	92.2 ± 219.2	140.1 ± 319.2	0.0269
IL-6sR (pg/mL)	3,375 ± 1,698	3,272 ± 1,166	0.0335
MIG (pg/mL)	446.8 ± 2,094	468.4 ± 1,454	0.039
BLC (pg/mL)	29.5 ± 51.1	41.1 ± 71.7	0.0456
IFNg (pg/mL)	102.4 ± 232.9	148.7 ± 321.7	0.0568
IL-2 (pg/mL)	835.8 ± 1,867	1,094 ± 2,538	0.0702
IL-1a (pg/mL)	168.1 ± 289.5	170.5 ± 380.1	0.1078
I-309 (pg/mL)	109.7 ± 239.9	114.4 ± 314.0	0.1119
IL-12p40 (pg/mL)	182.7 ± 551.4	280.2 ± 701.7	0.1539
TIMP-1 (pg/mL)	73,964 ± 56,059	75,086 ± 45,648	0.241
IL-5 (pg/mL)	108.2 ± 206.2	114.2 ± 174.4	0.338
TNF-RI (pg/mL)	7,060 ± 7,186	7,227 ± 6,154	0.3762
Eotaxin (pg/mL)	79.8 ± 101.5	71.3 ± 116.1	0.4235
TNF-RII (pg/mL)	24,984 ± 20,762	25,159 ± 23,732	0.4261
TNF-β (pg/mL)	5,861 ± 14,040	6,297 ± 12,228	0.4306
TNF-*α* (pg/mL)	561.1 ± 1,532	555.3 ± 1,335	0.4332
RANTES (pg/mL)	10,033 ± 7,212	11,364 ± 8,160	0.4615
Eotaxin-2 (pg/mL)	311.8 ± 229.2	285.5 ± 203.6	0.4999
IL-12p70 (pg/mL)	4.9 ± 18.0	5.9 ± 16.9	0.7133
PDGF (pg/mL)	3,742 ± 5,065	3,569 ± 5,229	0.842

## Discussion

The relationship between biomarkers and severity of COVID-19 has been discussed widely, including the stratification of inflammatory responses and organ-related involvement [24]. There have also been evaluations of the prognostic differences related to sex characteristics that displayed a positive correlation between males having a higher risk of disease severity and fatality. This study uniquely evaluated patients in a rural hospital setting and the differences in clinical course and outcomes to identify environmental prognostic factors. Our case series also evaluated the differences in immune responses associated with age and sex.

Patient demographics, especially ages, were different from Centers for Disease Control and Prevention (CDC) data from June 2020 to March 2021, which indicates that the majority of hospitalized COVID-19 patients were between the ages of 18 and 64 years, whereas the ages in this study were 42 to 81 years [25]. Compared to self-care patients, hospitalized patients were more elderly in this study. National and West Texas race dispersions were similar with non-Hispanic White, non-Hispanic Black, and Hispanic comprising the majority of the population. Hospitalization rates were split evenly between men and women nationally; however, in this study, the majority of non-critical inpatients and ICU patients were male. Due to the lack of racial diversity in the West Texas area, this study was not able to determine the differences in severity in different races. In this study, the majority of patients were obese; however, obesity was not correlated with the severity of the disease.

Common signs and symptoms seen in both national studies and this study included fever, chills, nausea/vomiting/diarrhea, loss of smell, shortness of breath, cough, headache, myalgias, sore throat, and abdominal pain. The most notable differences between the national population and this study population were the higher rates of patients experiencing shortness of breath, sore throat, abdominal pain, and altered mental status in this rural population. However, the national population experienced more coughs than patients in our study.

Among the molecular biomarkers, CRP, ferritin, LDH, D-dimer, and IL-10 showed a positive correlation with disease severity. These laboratory tests can potentially be used to develop a clinical algorithm for patient triage. CRP and LDH are elevated from tissue damage after a microbial infection. Ferritin and D-dimer are elevated during the inflammation. IL-10 is an anti-inflammatory cytokine associated with wound healing responses. We also observed that while almost all inflammatory markers were positively correlated, there was a negative correlation trend between PDGF and these immune markers. PDGF plays a role in angiogenesis and stimulation of cell cycle entry, processes that are necessary for tissue repair. Our study results suggest that COVID-19 leads to not only inflammation but also tissue damage and initiation of a tissue repair response that may be impaired.

We found significant differences in immune response markers in older patients ≥65 years compared to younger patients (<65 years), although there does not appear to be a distinct pattern. Some inflammatory molecules were higher while others were lower. Thus, while age seems to affect overall host immunity, the precise mechanisms are unclear.

We also identified molecular changes indicative of a more robust immune response in females which may be linked to the role of sex hormones in regulating immune cell functionality. We found that almost all immune modulatory molecules were higher in females than males, and most of these were cytokines involved in macrophage activation, immune cell recruitment (i.e., chemotaxis), and propagation of inflammation and T-cell responses. The higher levels in females support that there is a stronger immune response to SARS-CoV-2, which could partially explain the sex differences in COVID-19 morbidity and mortality. Estrogen plays a role in enhancing humoral immunity, which is responsible for the production of virus-neutralizing antibodies, while testosterone and androgens can act suppressively on the immune system, which may heighten susceptibility to COVID-19 pathogenesis in males [26-28].

A limitation of this study is that we only assessed patients who presented to the hospital, while the majority of people with COVID-19 have disease resolution after self-care at home or treatment in the outpatient setting. Hence, our sample likely represents those with more severe symptoms and more comorbidities. Another limitation is that not all COVID-19 patients who presented to the study site had adequate specimens for us to collect and these patients were not included in the immune protein microarray analyses. Finally, while we saw intriguing differences between older and younger patients, and between males and females, with regards to cytokine, chemokines, and other immune responses, some of these differences did not reach statistical significance likely due to the small sample size and heterogeneity in the sample population. Additional studies are warranted to more rigorously probe the age and sex differences in immune response to COVID-19 and dissect the underlying mechanisms.

## Conclusions

The clinical characteristics of this rural cohort of hospitalized patients differed somewhat from nationally reported data. Further studies focused on rural populations are needed to clarify the contribution of social, environmental, and healthcare access factors. We characterized 40 immune molecules and identified distinct age and sex-associated patterns of immune responses. These differences in immune functionality also have the potential to impact vaccine and treatment efficacy. Further studies are warranted to identify the underlying mechanisms and determine how this impacts COVID-19 pathogenesis and disease prognosis.
